# Spinal Cord Ischemia after Endovascular Repair of Infrarenal Abdominal Aortic Aneurysm: A Rare Complication

**DOI:** 10.1155/2011/954572

**Published:** 2011-06-16

**Authors:** George N. Kouvelos, Nektario Papa, Christos Nassis, Nikolaos Xiropotamos, George Papadopoulos, Miltiadis I. Matsagkas

**Affiliations:** ^1^Department of Surgery, Vascular Surgery Unit, Medical School, University of Ioannina, 45500 Ioannina, Greece; ^2^Intensive Care Unit, Medical School, University of Ioannina, 45500 Ioannina, Greece; ^3^Department of Anesthesiology, Medical School, University of Ioannina, 45500 Ioannina, Greece

## Abstract

Neurologic deficit secondary to spinal cord ischemia after elective infrarenal, endovascular aneurysm repair (EVAR), consists a rare and rather disastrous complication. The etiology of such neurologic complication seems to be multifactorial, making this event unpredictable and foremost unpreventable. We report a case of paraparesis and bladder dysfunction that occurred immediately after the EVAR procedure. Prompt management by conservative or invasive methods seems to be important for the reversal of the neurologic deficit and the optimization of patient's outcome.

## 1. Introduction

Neurologic complication due to spinal cord ischemia (SCI) after elective infrarenal abdominal aortic aneurysm (AAA) repair consists a rare and rather disastrous event. Szilagyi estimates the incidence of this complication to be approximately 1 case in 400 after open AAA repair and 1 case in 5000 after arterial reconstruction for aortoiliac occlusive disease [1]. The incorporation of EVAR into the vascular surgeon's armamentarium mainly during the last decade has changed the therapeutic perspective of AAA, as well as the perioperative complications observed. SCI has been occasionally seen after open repair but has rarely been encountered after EVAR. This report details a case of neurologic deficit in a patient treated electively for AAA by endoluminal means. 

## 2. Case Report

A 70-year-old man with a history of coronary artery disease, chronic obstructive pulmonary disease, and hyperlipidemia was referred to our department for evaluation of a 5.5 cm AAA. The patient had previously undergone a 3 vessel coronary artery by-pass grafting and was under antiplatelet therapy with acetylsalicylic acid (320 mg/day). The anatomic configuration of the AAA fulfilled the requirements for EVAR. The infrarenal neck of the aneurysm was reverse-tapered, 12 mm in length, and 26 mm to 29 mm in diameter. Due to these anatomic characteristics, a graft with suprarenal fixation was selected. Under general anesthesia an endovascular bifurcated graft (Talent, Medtronic Vascular AVE, Medtronic Europe SA, Route du Molliau, Switzerland) of 32 mm in main body diameter, 16 mm in limb diameter, and 15.5 cm in length was implanted. Due to the reverse-tapered shape of the aneurysm neck, the graft sustained a disposition 6 mm distally leading to an endoleak type I formation. Despite the application of a balloon inflation in the aneurysm neck, the endoleak did not resolve, so a proximal aortic cuff 34 mm in diameter was additionally deployed. Balloon remodeling was performed to the proximal and distal landing zones as well as to all overlapping sites according to the manufacturer's recommendations. Completion angiography revealed adequate graft interposition with exclusion of the aneurysm sac, maintenance of internal iliac arteries' perfusion, and no signs of endoleak. Total operative time was approximately 95 minutes. The patient had no intraoperative complications and remained hemodynamically stable throughout the operation.

Immediately after the procedure, the patient complained for slight global pain in both legs without any moving disability. The same evening as the patient was trying to mobilize, he experienced gait difficulty. Neurologic examination revealed bilateral lower extremity distal paraparesis involving mostly the posterior tibial group of muscles with decreased sensation mainly at 3-4 lower sacral segments on pinprick and sparing light touch. Bladder function was also lost. Urgent magnetic resonance imaging did not reveal evidence of cord compression, hematoma, or infarction. Immediately aggressive diuresis treatment was started. Intravenous solumedrol (5.4 mg/Kg bolus and 30 mg/kg drip) and mannitol (100 mL of 20% solution bolus, followed by 50 mL 4 times daily) were administered. Blood pressure was optimized with intravenous fluids, and mean arterial pressure was kept above 90 to 100 mm Hg. The aggressive diuresis treatment was continued for three days and moderate improvement was noted at neurological examination. The patient was discharged at the 7th postoperative day with no further complications. He followed a long rehabilitation programme and at the one-year followup has regained nearly fully walking ability but retained permanent bladder incontinence. No aneurysm related complications were recorded.

## 3. Discussion

Spinal cord ischemia has been reported after EVAR either as an immediate or a delayed finding [2–6]. The analysis of the EUROSTAR database including 2862 patients who had undergone EVAR found an incidence of 0.21% for SCI [7]. Peppelenbosch et al. demonstrated a much higher incidence after endovascular repair of ruptured AAA [8]. In their report SCI occurred in 11.5% of 35 patients after the deployment of an aorto-uni-iliac (AUI) device. In 2001, Rockman et al. reported the first two cases of lower extremity paraparesis subsequent to endovascular management of AAA [2]. In this report the second case was actually an attempted endovascular repair, since difficulties with the device's deployment led to standard open repair, thus raising the possibility that the neurologic event may have occurred at the time of the open repair. Bajwa et al. reported bilateral lower extremity sensory and motor loss with no bladder dysfunction after the deployment of an AUI device, while Lioupis et al. reported paraplegia in a patient with complex iliac anatomy, necessitating covering of one and reconstruction of the other hypogastric artery during a five-hour procedure [3, 4]. 

There are also some reports of patients emerging from elective surgery intact and then lately during the postoperative period developing delayed SCI. Kwok et al. reported delayed SCI presenting approximately two days after the procedure [5]. The patient demonstrated bilateral weakness and numbness with bowel and bladder incontinence, while the graft was extended to both external iliac arteries after embolization of both hypogastric arteries.

The process leading to spinal cord ischemia after elective management of infrarenal AAAs has not yet been fully understood. Factors that may contribute to spinal cord ischemia after open AAA repair include prolonged aortic occlusion, intraoperative hypotension, atheromatous embolization, interruption of the great radicular artery (artery of Adamkiewicz), or collateral circulation (internal iliac arteries-lumbar arteries) [1–3]. Given that EVAR consists a minimally invasive method of treatment of AAA, the last two of the above-mentioned factors seem to be mostly suited with the etiology of a neurogical complication.

Atheroembolization consist a well-known complication of endovascular surgery that may lead to SCI. Rockman et al. reported two cases of paraplegia as a result of atheroembolization of spinal cord after successful or attempted endovascular management of AAA [2]. In these patients, the extensive manipulation of catheters and other devices in severely atherosclerotic vessels resulted in trauma sufficient enough to cause dissemination of embolic material. In the EUROSTAR registry, factors associated with intraoperative microembolization included long procedure time (>150 minutes), extensive intravascular handling, and preoperative or perioperative embolizations of the hypogastric and lumbar arteries [4, 7]. In our case the necessity of balloon dilatations and to the deployment of the proximal aortic cuff may have raised the possibility of atheroembolization. The improvement of endovascular devices by reduction of their profile in combination with the improvement of the vascular surgeon's manipulation technique could be beneficial and may eventually lead to a reduced risk for atheroembolization in the future.

According to the interruption of collateral circulation, the deployment of an aortic stent graft for the exclusion of the aneurysm sac always leads to interruption of blood supply to the inferior mesenteric artery and all infrarenal lumbar arteries. Although these arteries could contribute to spinal cord blood supply, their occlusion could not solely explain SCI, as otherwise one would expect a much higher incidence of such complication. Indeed, as it was mentioned previously the analysis of the EUROSTAR database found an incidence of 0.21% for spinal cord ischaemia [7]. Curiously, the estimated occurrence is similar for either open or endovascular aneurysm treatment [1, 7].

Alternatively, the occlusion of the internal iliac artery could potentially induce spinal cord ischemia especially in patients whose spinal cord perfusion is dependent on the pelvic circulation. Bratby et al. in a recent report assessed the outcomes of bilateral internal iliac artery embolisation prior to EVAR and found 3% incidence rate of paraparesis [9]. On the other side, Mehta et al. in an older report actually argued the role of bilateral hypogastric artery embolization as they report no neurologic deficits in 48 patients treated for aortoiliac aneurysm disease with intentional bilateral IIA interruption [10]. In our case as confirmed in both the intraoperative completion angiography, as well in the postoperative CT angiography, there was no occlusion of the IIA, so this mechanism could not be considered to have contributed to the neurologic deficit.

The Adamkiewicz artery (AKA) supplies most of the blood to the anterior spinal artery, which perfuses the anterior two thirds of the spinal cord. AKA originates variably between T5 and L3 and from the left side in 75% of cases [11]. In 7.5% of cases also it arises from L1-L2 level, while in 0.8% of cases it originates from L3 level [12]. The main renal artery in 75% of the general population originates from the level of L1-L2 intervertebral disc, while the other 25% originates somewhere between the lower end plates of T12 and L2 [13]. Based on these data, it is clear that the interpretation between AKA's orifice and the infrarenal segment of the aorta covered by the graft is very limited. However, the deployment of a graft with suprarenal fixation may theoretically raise the possibilities of partially covering AKA's origination. On the other hand, the identification of the AKA's orifice is not always achievable. Melissano et al. using open source software and low cost hardware managed to identify the AKA origin in 51 (76,1%) of the total 67 patients with thoracic or thoracoabdominal disease. By reviewing the literature, they found that recognition of the AKA was achieved in 466 of 555 cases (83,9%) and that in 83.3% of the cases the AKA originated from a left intercostal artery [12]. Nevertheless, it is yet uncertain if preoperative knowledge of the AKA location, especially in AAA management, could contribute to an effectively different strategy to avoid spinal cord ischemia.

Our current comprehension of neurologic deficit after aortic surgery is mainly acquired from the experience of descending thoracic and thoracoabdominal aortic repair. The goal of the treatment is to augment spinal cord perfusion pressure and reduce oedema. The therapeutic strategy includes cerebrospinal fluid (CSF) drainage, hypothermia, steroids, and arterial pressure augmentation. CSF drainage has been shown to offer significant neurologic protection perioperatively in patients undergoing TAAA repair. Hnath et al. compared two groups of 121 patients undergoing endovascular thoracic aortic repair with or without preoperative preventive CSF drainage, and found significantly less prevalence of SCI in the CSF drainage group [14]. From 121 patients 5 developed clinical symptoms of paraparesis, and after CSF drainage placement 3 demonstrated marked clinical improvement [14]. Additionally, Cheung et al. reported two cases of paraplegia after TEVAR fully recovered after arterial pressure augmentation alone and 3 cases recovered after combination of arterial pressure optimization and CSF drainage [15]. On the other hand, the current knowledge for the optimal management of paraplegia after EVAR for infrarenal AAA has mainly derived from case reports. Lioupis et al. noted partially neurologic recovery after CSF drainage, while Bajwa et al. managed to accomplish with CSF drainage complete resolution of neurological deficit in a patient underwent EVAR [3, 4]. However, spinal fluid drainage is not always free of complications, as Wynn et al. reported a rate of neurologic deficit of 1% and even a mortality rate of 0.6% [16]. The fact that our patient was under high dosage of antiplatelet drug prevented us from performing CSF drainage. In our case the combination of steroids and arterial pressure augmentation, the least invasive management strategy, led eventually to moderate improvement of the neurological state of our patient. 

In summary, neurologic deficit after EVAR for infrarenal AAA consists a serious and rather disastrous complication. Its multifactorial etiology makes this event unpredictable and in the majority of cases unpreventable. Currently, there are no clear data that may suggest the optimal (conservative or invasive) treatment for the management of SCI after EVAR. However, the preservation of collateral circulation whenever possible, a meticulous surgical planning, the improvement of endovascular techniques, and devices and the maintenance of optimal blood pressure perioperatively could act as preventive measures leading to optimization of patient's outcome. 
